# Chromosomal Aberrations Associated with Clonal Evolution and Leukemic Transformation in Fanconi Anemia: Clinical and Biological Implications

**DOI:** 10.1155/2012/349837

**Published:** 2012-05-23

**Authors:** Stefan Meyer, Heidemarie Neitzel, Holger Tönnies

**Affiliations:** ^1^c/o Young Oncology Unit, Department of Paediatric and Adolescent Oncology, Christie Hospital, Wilmslow Road, Manchester M20 6XB, UK; ^2^Stem Cell & Leukaemia Proteomics Laboratory, School of Cancer and Enabling Sciences, Faculty of Medical and Human Sciences, University of Manchester, Manchester Academic Health Science Centre, Manchester, UK; ^3^Department of Paediatric Oncology, Royal Manchester Children's Hospital Oxford Road, Manchester M13 9WL, UK; ^4^Institute for Medical and Human Genetics, Charité Universitätsmedizin Berlin, Germany, Campus Virchow-Klinikum, Augustenburger Platz 1, 13353 Berlin, Germany; ^5^Gendiagnostik Kommission, Robert Koch Institut, DGZ-Ring 1, 13086 Berlin, Germany

## Abstract

Fanconi anaemia (FA) is an inherited disease with congenital and developmental abnormalities, bone marrow failure, and extreme risk of leukemic transformation. Bone marrow surveillance is an important part of the clinical management of FA and often reveals cytogenetic aberrations. Here, we review bone marrow findings in FA and discuss the clinical and biological implications of chromosomal aberrations associated with leukemic transformation.

## 1. Introduction

Fanconi anemia (FA) is an inherited disease with bone marrow failure, variable congenital and developmental abnormalities, and extreme cancer predisposition. The most common malignancies in FA are myeloid leukemia and squamous cell carcinoma. On a cellular level, FA is characterized by chromosomal instability and cross-linker sensitivity, which is the diagnostic hallmark of FA. For diagnostic testing, this is determined by demonstration of hypersensitivity to mitomycin C (MMC) or diepoxybutane (DEB) of patient derived peripheral blood cells or fibroblasts [1–3]. FA cells also display hypersensitivity to proapoptotic stimuli of certain cytokines, such as TNF-*α* and IFN-*γ*, which has been implicated in haematological manifestations of FA [4–6]. Cell cycle analysis of FA cells shows a characteristic arrest in the G2 phase, which is exacerbated by exposure to MMC [7–9]. This clinical and cellular phenotype results from a defect in a DNA damage response (DDR) pathway (FA/BRCA pathway), in which FA and associated proteins interact. So far, 15 FA genes* (FANCA, FANCB, FANCC, FANCD1/BRCA2, FANCD2, FANCE, FANCF, FANCG, FANCI, FANCJ, FANCL, FANCM, FANCN/PALPB2, FANCO/RAD51C,* and* FANCP/SLX4)* have been identified that can be mutated in FA [2, 10–12], of which *FANCA*, *FANCG*, and *FANCC* are the most commonly mutated genes in studied FA populations [2]. Importantly, the discovery that mutations in *BRCA2 *causes FA in the subgroup FA-D1, which comprises less than 5% of all FA patients, linked the FA DNA damage response pathway to hereditary breast and ovarian cancer (HBOC) [13, 14]. Hematopoiesis in the bone marrow (BM) is the most commonly affected organ system in FA, and most FA patients will develop clinically relevant hematological complications in their first or second decades of life [15]. BM complications of FA can manifest with hypoplasia, often initially being limited to thrombocytopenia in peripheral blood counts, or general aplasia. When the diagnosis of FA is made, which might happen with considerable delay, bone marrow appearances can already be more advanced and consistent with myelodysplasia. In FA, this often presents as refractory cytopenia with multilineage dysplasia, with or without excess of blasts on morphologic evaluation. Common morphologic abnormalities on bone marrow examination include irregular nuclear contours, budding nuclei, and karyorrhexis [16]. In some patients, the diagnosis of FA is only made on presentation with overt myeloid leukemia. How common undiagnosed FA presents with AML is not known, but FA should be considered especially in young patients with AML, even in the absence of sometimes only subtle congenital malformations such as short stature and microcephaly, in particular when excess toxicity or prolonged aplasia after chemotherapy of extreme toxicity is encountered [17–19]. Less than ten cases of lymphoblastic leukemias have been reported in FA, which have been mostly of T-lineage, and appear to be limited to patients with mutations in *FANCD1/BRCA2* and *FANCD2 *[19–21].

Given the high incidence of hematological complications of FA, BM surveillance for morphological and cytogenetic changes makes an important contribution to the clinical management of FA patients. With improved and more sensitive methods for the detection of chromosomal aberrations over the last decade, and better understanding of clinical implications of BM cytogenetic findings in general for preleukemic changes and the diagnosis and management of hematological malignancies, FA bone marrow surveillance provides important information for clinical decision making. In addition, clonal evolution and associated chromosomal aberrations in FA are important for understanding malignant transformation in general and therefore wider implications. Here, we review bone marrow chromosomal aberrations in FA and discuss the clinical and biological implications.

### 1.1. Bone Marrow Surveillance: Clinical Aspects for Patient Management

In view of the relative infrequency and clinical variety of FA, no evidence-based data exist on how and how frequent bone marrow should be surveyed in FA patients. In view of variable practice, a recent survey carried out in the UK (S. Meyer, unpublished data), confirmed broadly the recommended practice, in that FA patients with normal blood counts should have at least a yearly assessment of bone marrow morphology and cytogenetics [3, 22]. If there is evidence of bone marrow failure, most centers would consider increasing the frequency and monitor more closely for appearance and evolution of chromosomal aberrations. For the initial management planning at diagnosis, it is worth considering that absent radius and a severe phenotype is statistically associated with earlier bone marrow failure, and that of the most commonly mutated genes, *FANCG* and *FANCC* have a statistically higher incidence of early hematological complications than *FANCA* [23]. However, BM failure is a common presentation of FA caused by mutations in all complementation groups. Surveillance for FA-associated BM manifestations should include morphology and assessment of cellularity as well as cytogenetic evaluation [3]. The cytogenetic evaluation should include conventional karyotyping. Importantly, however, cytogenetic analysis should specifically include investigations for FA-characteristic chromosomal aberrations as outlined below. Only with the application of more sophisticated cytogenetic methodologies the incidence and significance of FA-specific aberrations can be determined. These would include routine application of or/and comparative genomic hybridization (CGH) or more sensitive whole genome analysis such as array CGH, in addition to fluorescent in situ hybridization (FISH), targeting FA-specific chromosomal gains and losses on a single cell level.

### 1.2. Spectrum of FA-Specific BM Chromosomal Aberrations

**Figure 1:**
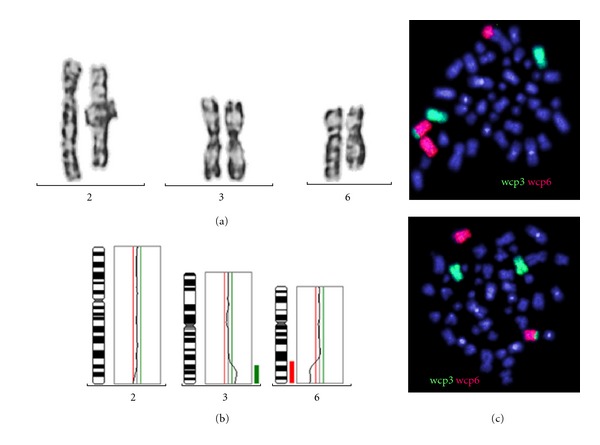
FA-associated 3q aberrations. (a) conventional cytogenetics: outcuts of chromosome 2, 3, and 6 showing additional material at 2q, normal chromosomes 3, and an apparent deletion of 6q. (b) the conventional CGH shows a slight deviation at 2q, a gain of 3q25 to 3qter (enh), and a loss of material from 6q23 to 6qter (dim). (c) the FISH with whole chromosome paints wcp3 und wcp6 demonstrates two cell lines: one with material of chromosome 6 translocated to 2q and with material of 3 translocated to 6q; another cell line which carries only the translocation of material of chromosome 3 to 6q. Thus, the apparent deletion detected by conventional cytogenetics proved to be not a sole deletion of 6q but in addition a unbalanced translocation of 3q onto 6q. In addition, the patient had a monosomy 7 (data not shown). The karyotype according to ISCN 2009 in bone marrow cells was 45,XY,-7[2]/45,der(6)(6pter**→**6q22::3q25**→**3qter),-7[27]/45,der(2)(2pter**→**2q37::6q22**→**6qter),der(6)(6pter**→**6q22::3q25**→**3qter),-7[8].

Clonal bone marrow aberrations in individuals affected by FA were first reported as far back as the 1970s and early 1980s, when several studies recognized cytogenetic abnormalities on bone marrow examination of FA patients, many of them noting a high frequency of monosomy 7, detected by conventional karyotyping [25, 26]. The clinical observation that the detectability of chromosomal aberrations in bone marrow aspirates of FA patients can vary over time, with clones becoming transiently undetectable, has led to an underestimation of clinical relevance of chromosomal aberrations in FA [27]. In addition, the absence of nonrandom chromosomal rearrangements that are frequently found in AML in particular in childhood, has delayed the recognition and the clinical and prognostic significance of specific aberrations frequently seen in FA [24]. A better understanding of the clinical relevance and biological implications of chromosomal aberrations in FA was achieved over the last decade by analysis of larger case series and the application of modern molecular cytogenetic technologies in addition to conventional karyotyping [16, 28–31]. This has led to the identification and delineation of specific patterns of chromosomal aberrations in FA. In contrast to aberrations seen in sporadic AML in childhood, these are characteristically unbalanced, with gains and losses of chromosomal material during clonal evolution. Frequent for FA are gains of the chromosomal regions 1q and 3q, as illustrated in Figure 1, and partial or complete loss of chromosome 7 [16, 29–33]. Of these, 3q gains are in particular characteristic of FA. By studying larger numbers of FA patients sequentially, not only the high specificity for FA became evident, but also the clinical implication of 3q gains, of which occurrence indicate transformation to MDS and AML [30, 31]. In four independent studies, two from Europe and two from North America, the association of 3q gains with progression to or presence of FA-related myelodysplasia was confirmed [16, 29–31]. Importantly, gains involving 3q are only rarely seen in BM from non-Fanconi patients [34–36], while balanced chromosomal aberrations, such as inversions or translocations involving the 3q are well documented in myeloid malignancies from non-FA patients, in particular in adults [35, 36]. Therefore, cytogenetic detection of 3q gains in apparently sporadic cases of MDS or AML would indicate testing for FA. The impact on gene expression resulting from FA-specific gains in the area of common amplification, 3q26-3q29, has only recently been studied and point to an important role of the transcriptional regulator *EVI1* (ecotropic viral integration site 1) for leukaemic transformation in FA [37, 38]. Another frequently observed aberration in FA is gain of chromosomal material at 1q. This aberration can also be present in morphologically relatively normal BM and is a finding also in non FA-hematological diseases. Its presence is often the sole finding in the early stages of clonal evolution and can persist for years, but also occurs frequently with 3q gains and other aberrations. Chromosomal aberration involving chromosome 7 include -7/-7q, which, as in the non-FA population, is significantly correlated with more advanced dysplasia and commonly part of a clone with a more complex karyotype that frequently also shows gain of 3q material [39]. Sequential analysis of clonal progression in FA has revealed that 3q-gains often precede changes involving partial or whole loss of chromosome 7 [30, 37]. Another more recently recognized frequent finding in FA-associated clonal evolution is 11q-in advancing FA-associated MDS. This lesion occurs in FA frequently with a more complex karyotype that also shows 3q gain and/or -7 [31]. The recent detection of involvement of the *RUNX1* locus at 21q in FA-associated genomic abnormalities, which in all cases were associated with advanced MDS [31], has also some important biological implications for the understanding of clonal evolution with FA, which is discussed below. Gains and losses of chromosomal material can also involve other chromosomes, but not with the same FA-specific patterns as for 3q. Balanced translocations have been described in FA, and occur usually as part of more complex clonal rearrangements. Single cases also had involvement of the 3q region as well a single report of an 11q23 translocation [21, 38]. Importantly, common balanced nonrandom chromosomal rearrangements that are seen in AML, such as t(8; 21) or inv(16) translocations, have never been reported in FA [24].

### 1.3. Clonal FA-Associated Bone Marrow Aberrations: Clinical Implications

Hematological complications are the most common manifestation of FA. Over the last three decades according to large studies carried out in North America and Europe, the cumulative incidence of any hematological abnormality in FA approaches 90%, and the cumulative incidence of leukemia has been approximately 30% by 40 years of age [20, 23]. Therapeutically, options to treat bone marrow failure in FA are limited to interventions with growth factors and androgens in order to improve peripheral blood counts [3], but this does not alter the high risk of leukemic transformation. The most important management decision for hematological complications of FA is when and how to proceed to hematopoietic stem cell transplantation (HSCT). The outcome of HSCT in FA has improved dramatically [40, 41], and the incidence and survival patterns quoted above, as well the clinical course of FA are changing accordingly [23]. Many centers would elect HSCT for FA in the presence of significant hematological abnormalities and availability of a suitable donor. Cytogenetic information would certainly inform decision making, and the presence of chromosomal aberrations, in particular those associated with high risk of malignant transformation would potentially justify a more aggressive approach that could include partly mismatched donors [41, 42]. Leukemia in FA is very difficult to treat. From sparse published data of relatively few reported cases, overt leukemia in FA is associated with poor prognosis and short survival. Conditioning regimes for FA have empirically been tailored for the intrinsic chemosensitivity of FA patients and are increasingly based on fludarabine with low-dose cyclophosphamide. There is little evidence that the presence of BM cytogenetic aberrations should influence conditioning regimes, as long as there is no evidence of overt leukemia. However, numbers of reported cases are very small [40, 41, 43], and several cases of leukemic relapse after HSCT have been reported, of which intriguingly one was from donor cells [44].

### 1.4. FA-Characteristic Chromosomal Aberrations: Implications for Malignant Transformation

The emergence of characteristic patterns of chromosomal aberrations in FA has relevance for the management of FA patients. Detection of chromosomal aberrations that confer a high risk of transformation to MDS and AML warrants a more aggressive approach in order to prevent leukemia development. However, the study of chromosomal aberrations in this disorder has some more generally relevant implications, giving insight of secondary events in clonal evolution arising associated with an inherited defect in the DNA damage response. The FA-characteristic clonal evolution with dominance of chromosomal gains and losses is likely to be a specific result of the disruption of the FA/BRCA pathway and at least partially caused by FA-related unresolved DNA damage during S phase. This unresolved DNA damage is thought to lead to the FA-specific G2 arrest and could lead to the accumulation double-strand breaks and switch to more error-prone repair by nonhomologous end joining. Indeed, nonhomologous end joining is largely efficient in FA cells in contrast to homologous repair, which is grossly impaired in FA [45–47]. However, this possible and extremely simplified explanation could only partly explain the occurrence of aberrations, but not characteristic patterns of chromosomal aberrations involving typically 3q and 1q. The striking overrepresentation of 1q and 3q could imply that these chromosomal regions are particularly susceptible to FA/BRCA disruption-associated damage. An alternative or additional explanation would be that resulting genetic changes confer a growth advantage, possibly in particular in the presence of a defect in the FA/BRCA pathway. Cytogenetic analysis of sporadic AMLs that occur in a comparable age group of older children and young adults shows some marked differences when compared with FA-associated leukaemic transformation, exemplified by the rarity of any 3q aberrations in less than 5% of childhood the AML in the NPO studies and the MRC trials [48, 49], and exceedingly rare findings of gains of chromosomal material in this region, implying an alternative pathogenesis of FA-associated leukaemic transformation. The FA-characteristic 3q gains nearly always harbor one of the most aggressive leukemogenic oncogenes, *EVI1*, which was first detected to be amplified and overexpressed as an initial event in FA-derived AML transformation in patient material and cell lines from a patient with biallelic FANCD1/BRCA2 mutations [16, 31, 38] and subsequently shown to result in overexpression of *EVI1 *[37]. This suggest that FA-associated leukemia shares its biology with one of the most aggressive forms of sporadic AML [35, 36]. Intriguingly, *EVI1* overexpression in childhood AML, which has been detected in approximately 10% of cases, is normally not a result of chromosomal rearrangements of the 3q region, but appears to be associated other chromosomal rearrangements and is not of the same prognostic relevance as *EVI1* overexpression resulting from chromosomal rearrangements [50]. The other specific gene that appears to be targeted by FA-associated chromosomal rearrangements is *RUNX1, *which points to the question as to how chromosomal rearrangements in FA promote leukemic transformation. One important observation comes from studies with FANCC -/- mice. Leukaemic clones in bone marrow of these mice that were outgrown under the selective pressure of TNF-*α* showed abrogated cytokine sensitivity occurring together with chromosomal aberrations [51]. In addition, analysis of patient derived BM cells of FA patients with chromosomal aberrations led to the detection of an attenuated cellular FA phenotype. Cells with this phenotype maintained lack of FANCD2 ubiquitination associated with FA core complex gene mutations, and MMC hypersensitivity, but did not display the FA-specific G2 arrest on cell cycle analysis [28]. It will be important to explore to what extent and by which mechanism individual or combined the genetic changes associated with leukemic transformation in FA modulating the cellular FA phenotype. Taken together, these observations point to a modulatory effect mediated by chromosomal aberrations on the cellular FA phenotype, which is likely to be of general relevance for oncogene-mediated malignant progression and the DNA damage response [52].
